# Identification of G1-Regulated Genes in Normally Cycling Human Cells

**DOI:** 10.1371/journal.pone.0003943

**Published:** 2008-12-15

**Authors:** Maroun J. Beyrouthy, Karen E. Alexander, Amy Baldwin, Michael L. Whitfield, Hank W. Bass, Dan McGee, Myra M. Hurt

**Affiliations:** 1 Department of Biomedical Sciences, College of Medicine, Florida State University, Tallahassee, Florida, United States of America; 2 The Channing Laboratory, Brigham and Women's Hospital and Department of Medicine, Harvard Medical School, Boston, Massachusetts, United States of America; 3 Department of Genetics, Norris Cotton Cancer Center, Dartmouth Medical School, Hanover, New Hampshire, United States of America; 4 Department of Biological Science, Florida State University, Tallahassee, Florida, United States of America; 5 Department of Pharmacology and Toxicology, Dartmouth Medical School, Hanover, New Hampshire, United States of America; 6 Department of Statistics, Florida State University, Tallahassee, Florida, United States of America; Georgia Institute of Technology, United States of America

## Abstract

**Background:**

Obtaining synchronous cell populations is essential for cell-cycle studies. Methods such as serum withdrawal or use of drugs which block cells at specific points in the cell cycle alter cellular events upon re-entry into the cell cycle. Regulatory events occurring in early G1 phase of a new cell cycle could have been overlooked.

**Methodology and Findings:**

We used a robotic mitotic shake-off apparatus to select cells in late mitosis for genome-wide gene expression studies. Two separate microarray experiments were conducted, one which involved isolation of RNA hourly for several hours from synchronous cell populations, and one experiment which examined gene activity every 15 minutes from late telophase of mitosis into G1 phase. To verify synchrony of the cell populations under study, we utilized methods including BrdU uptake, FACS, and microarray analyses of histone gene activity. We also examined stress response gene activity. Our analysis enabled identification of 200 early G1-regulated genes, many of which currently have unknown functions. We also confirmed the expression of a set of genes candidates (*fos*, *atf3* and *tceb*) by qPCR to further validate the newly identified genes.

**Conclusion and Significance:**

Genome-scale expression analyses of the first two hours of G1 in naturally cycling cells enabled the discovery of a unique set of G1-regulated genes, many of which currently have unknown functions, in cells progressing normally through the cell division cycle. This group of genes may contain future targets for drug development and treatment of human disease.

## Introduction

Our studies of histone gene regulation have fostered a long term interest in events occurring in G1 phase and G1/S phase transition of the cell cycle [1]–[5]. This interest culminated in human genome-scale microarray experiments presented here. Human genome activity was examined in the early minutes of a new cell cycle in continuously cycling cells. Our approach was to conduct these studies in cells progressing naturally through the cell cycle with the goal of discovering genes whose G1 phase activity may not have been previously observed due to synchronization methods used.

To study events occurring in the cell division cycle, it is essential to be able to obtain synchronous populations of cells. Researchers have used different methods to achieve this goal. The most common technique used in the past was serum starvation [6], which arrested cells by limiting growth factors and other nutrients essential for cell growth in culture media. Although the response to serum does result in cells re-entering the cell cycle, it also results in a prominent wound-healing response [7]. The complex nature of the serum response means that distinguishing cell cycle genes from those involved in wound healing is very difficult. Other methods that attempt to avoid the serum response involve use of drugs to arrest cells at a definite stage of the cell cycle. Several methods that block cells in S phase include the DNA synthesis inhibitors such as aphidicolin, an inhibitor of DNA polymerase β [8], hydroxyurea [9], an inhibitor of ribonucleotide reductase [10], and excess thymidine which inhibits deoxycytidine deaminase. Other methods arrest cells in mitosis with drugs such as nocodazole [11] and colchicine [12] which have been used to disrupt the formation of the mitotic spindle by inhibiting microtubule polymerization, blocking the cells in G2/M, prior to entry into mitosis. All of these methods stress the cells and therefore alter the cellular response under investigation.

Our interest in studying events occurring in G1 and early S phase led us to develop a robotic mitotic shake-off apparatus. Since serum starvation blocks cells in G0 and they reenter the cycle in late G1 upon readdition of serum to the growth medium [13], [14], any regulatory events occurring early in G1 of continuously cycling cells are missed. Similarly, nocodazole-blocked cells seemed inappropriate for our purposes. Since gene knock-out and knock-down studies of G1 and G1/S regulators have presented a complicated picture of regulation of entry into a new cycle [15]–[19], an alternative synchronization method such as mitotic selection might add to our understanding of gene regulation in this part of the cell cycle.

Here we describe a new approach to an old technique, mitotic selection, described years ago [20], [21]. We have improved upon the approach significantly with new ideas and instrumentation. We have also validated the technique of mitotic selection using the latest experimental technologies. Our large-scale automated technique allows significant insights into a key window of the cell division cycle not afforded by other commonly used approaches for synchronizing mammalian cells [9], [11], [12]. The use of a fully automated mitotic shake-off machine to obtain synchronous populations of normally cycling cells without the use of drugs is described. We show data utilizing immunocytochemistry, flow cytometry and microarray technology to prove that mitotic cells selected by mitotic shake-off enter the next cell cycle normally, without activating stress response genes. Because of the powerful tools available to study regulatory activities at the molecular level, mitotic selection is an ideal method to obtain synchronous populations of cells for the study of early events in the cell cycle. This approach has enabled the identification of new candidate genes involved in regulation of early G1 of the cell cycle.

## Results

We wanted to examine gene activity in early G1 phase of the cell cycle in human cells moving naturally from mitosis into a new cell cycle. Most of what is known about G1 phase regulation came originally from studies utilizing cells synchronized by serum starvation, contact inhibition, thymidine blocks or drugs [18]. Overexpression and gene knock-out or knock-down of potential or known regulators has demonstrated that most cyclins, cdks and inhibitors are not essential for viability or for G1/S phase transition in normally cycling cells [18]. It has become increasingly clear that transition from quiescence into late G1 phase is not the same as transition from mitosis into early G1 of a new cell cycle [18]. Moreover, mitosis to G1 phase transition in cells not continuously cycling (i.e., cells released from blocks in S or G2/M transition) may differ in genome-wide transcriptional activity. Thus, we took a different experimental approach, mitotic selection, to identify potential growth regulators in the first two hours of G1 in continuously cycling HeLa cells.

### Collection of Mitotic Cells by Selective Detachment

We used an automated mitotic shake-off machine to collect mitotic cells [3]. This procedure takes advantage of a physical phenomenon, that in late mitosis as cells prepare to divide, they round up and their attachments to the growth substrate are loosened. Vigorous shaking of the growth flask releases mitotic cells from the growth substrate into the culture medium, allowing selection of mitotic cells. Of the cells tested in our lab for mitotic selection, HeLa and CHO cells are perfectly suited for mitotic selection. These cells exhibit morphology that includes regular cell margins and spacing patterns when examined by the light microscope.

Mitotic cells were collected every 10 minutes into 250 ml-conical tubes and stored on ice. Since microtubule polymerization is inhibited at temperatures below 4°C [22], cells may be held in late telophase of mitosis on ice for short periods [21]. Before collecting mitotic cells, pre-experimental clearing shakes are performed to get rid of non-adhering and/or dead cells. Cells are then pelleted, resuspended in CO_2_-adapted media and returned to standard growth conditions. Ten to fifteen minutes after plating, the cells exit mitosis and proceed into a new cell cycle.

### Correlation of Cell Synchrony by BrdU Uptake and FACS Analysis

**Figure 1:**
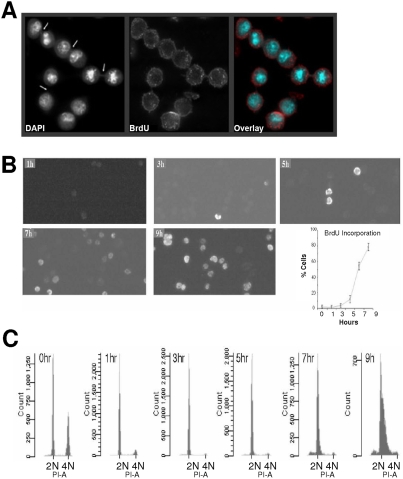
Synchrony in cells collected by mitotic selection. (A) Synchronous population of CHO cells 1 hour after mitotic selection. Images are displayed in grayscale for DAPI and BrdU uptake (Bromodeoxyuridine) and together as two-color overlay (cyan for DAPI and red for BrdU). The post-mitotic bridge between mitotic doublets is indicated by white arrow. Images were captured using a deconvolution microscope. Each image is a projection of 10 optical sections comprising a total thickness of 2.5 micrometer from the middle of the cell. Identical results are observed with HeLa cells [4]. (B) Entry into S phase by cycling HeLa cells. BrdU uptake by HeLa cells obtained by mitotic selection. 1→9 h indicate time after plating of mitotically selected cells. Graph shows percentage of cells incorporating BrdU at different time points after mitosis. A minimum of 300 cells were counted for each time point from at least eight fields of vision on the fixed slides. (C) FACS analysis of cells selected by mitotic shake-off. Mitotic HeLa cells were collected, plated and then harvested at specific time points. The cells were stained with propidium iodide (PI-A) and analyzed by FACS. BrdU incorporation in Figure 1B and FACS analysis were done in parallel. Each experiment was repeated twice.

Post-mitotic pairs of CHO cells, 1 hour after plating are shown in Figure 1A. Cytoplasmic bridges at the conclusion of cytokinesis are visible between some of the pairs of daughter cells. Bromodeoxyuridine (BrdU) incorporation is used to detect S phase entry and as these cells are in early G1 of the cell cycle, no BrdU incorporation is detected. Figure 1B shows microscopic fields of HeLa cells fixed at the indicated time after mitotic selection. S-phase cells incorporating BrdU and exhibiting fluorescent nuclei were counted in 8–10 fields for each time point and the results are shown graphically in Figure 1B. A very low number of cells enter S phase prior to 5 hours, indicating that cells prior to this time point are in G1 phase of a new cell cycle. The data presented in this figure reproduces the observations of others who have used manual or partially automated applications of the shake-off method [21], [23].

We also examined cell synchrony in mitotically selected cell populations by flow cytometry of propidium iodide (PI) labeled cells. Figure 1C shows an analysis of mitotically selected HeLa cells at different time points after plating, as they progress into a new cell cycle. At 0 hour (mitotic cells harvested by shake-off and directly analyzed), two populations of cells are present with either G1 (2N) or G2/M DNA content (4N). This likely results from cells in late telophase undergoing cell division during the moments under analysis (over 70%) and entering G1, while the cells which have not yet completed cytokinesis show 4N DNA content (less than 30%). At 1–3 hrs, nearly 100% of the cells are in G1 as evident by one DNA peak showing 2N DNA content. Cells enter S phase from 5–9 hrs. The right shift in the 2N peak becomes a shoulder in 7 hour and 9 hour samples and is indicative of cells in S phase of the cycle. The data shown here are evidence that these cell populations obtained by mitotic selection remain synchronized over the time course of our experiments.

### Correct Genomic Histone Gene Activity in the Cell Cycle in Mitotically Selected Cell Populations

We then examined the synchrony of mitotically selected cells by obtaining the gene expression profiles of the replication-dependent histone genes. The temporal regulation of expression of the histone gene family is well characterized [24]. Histone genes are classified as replication dependent and replication independent, with the replication-dependent (RD) genes undergoing up-regulation at the G1/S boundary of the cell cycle [2], [25]–[29]. Replication-independent histone (RI) genes show low, constitutive levels of expression across the cell cycle.

We used human genome-scale microarray analyses of RNA samples obtained from populations of cells progressing into the cell cycle after mitotic selection. The two genome scale microarray experiments are referred to here as *shake 1*, which includes RNA samples collected over a period of fourteen hours after mitotic selection at intervals of two hours (8 slide arrays) and *shake 2*, where samples were collected in fifteen-minute intervals over a period of two hours after mitotic selection (9 slide arrays). Thus, *shake 1* examines gene activity from mitosis to the midpoint and beyond of S phase. *Shake 2* is a close examination of the first two hours of G1. In a study that was published previously [5], a genome-scale analysis identified cell cycle-regulated genes in the human genome by identifying those genes with common expression patterns. RNAs were collected from HeLa cells synchronized by three different methods including double thymidine block, thymidine-nocodozole block, and mitotic selection. Those genes (>850) showing regulation in the cell cycle common to all three datasets were identified as cell cycle regulated genes [5]. This collaborative study included our total RNA samples which were obtained from cells synchronized by the mitotic selection technique, which we are identifying as *shake 1*.

**Figure 2:**
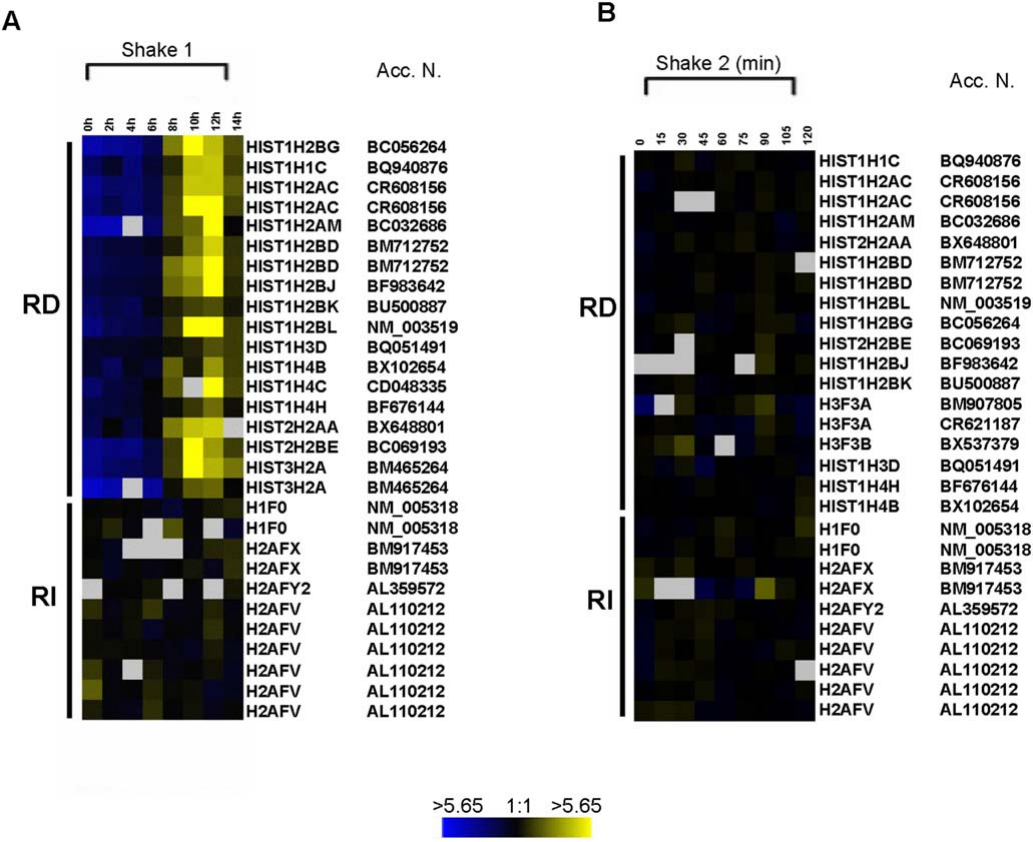
Gene expression profile of human histone genes on human genome-scale (29,000 genes) arrays. (A) The data shown here is from *shake 1* (14 h). (B) Data from *shake 2* (2 h). Refer to the text for detailed description of the experiment. The bars on the left separate the *replication-dependent* (RD) histone gene family from *replication-independent* (RI) histone genes. RD genes upregulation is indicated by a yellow color. Gray squares represent spots on the array with poor quality that were not considered in the analysis and did not make it to the final cluster image. The data presented here (A and B) is from two independent experiments, using two different sets of mRNAs and microarray slides. The color scale at the bottom indicates fold induction or repression in gene expression. The signal is a direct measure of relative abundance of mRNA sample from control and experimental samples. The gene symbols and accession numbers displayed are generated from SMD and S.O.U.R.C.E online tool. Gene symbols are represented close to the cluster image. The accession numbers (Acc. N.) are displayed far right to the image.

Here, we examined the expression of the replication-dependent histone genes in our datasets from *shake 1* and *shake 2*. Note that replication-dependent genes do not produce polyadenylated mRNA and are not in standard EST collections, but were deliberately included on these arrays. Figure 2A profiles the histone gene activity in *shake 1*. The RD histone gene activity shows 7–8 fold upregulation of these genes (e.g. *hist1h1c*, *hist1h2am*, *hist1h2bf*, *hist1h3d*, and *hist1h4c*) at the expected time, 6 hours and beyond, when the cells are entering S phase of the cell cycle. The replication-independent genes such as *h2afx* and *h2av* show a relatively constant expression profile across all time points (0 to 14 hours). Figure 2B includes expression data from *shake 2* which includes most of the histone genes shown in Figure 2A (*shake 1*). All of the RD histone genes on the *shake 2* arrays show no evidence of upregulation as expected for cells in early G1 phase. Upregulation of the RD genes does not occur at this early point in the cell cycle, which spans from telophase through early G1. The RD histone gene expression data observed in *shake 2* correlates very well with histone gene activity observed in the early time points of *shake 1*, i.e., 0 h and 2 h (compare Figure 2A and B). To identify specific cDNAs of interest for gene activity profiles shown in Figures 2–5, more information is provided in the Materials and Methods section.

**Figure 3 pone-0003943-g003:**
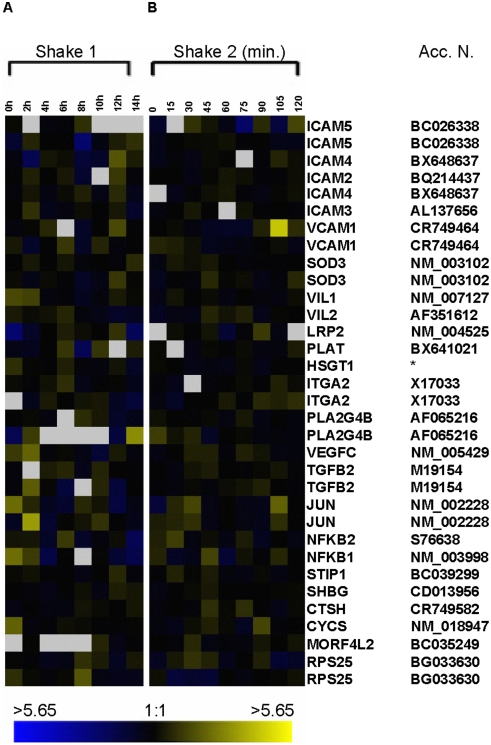
Gene expression profile of some mechanical stress-induced genes. The data included is from (A) *shake 1* and (B) *shake 2* experiments. The microarray analysis was performed as in Figure 2, but using genes identified in stress-response studies [30]–[33].

**Figure 4 pone-0003943-g004:**
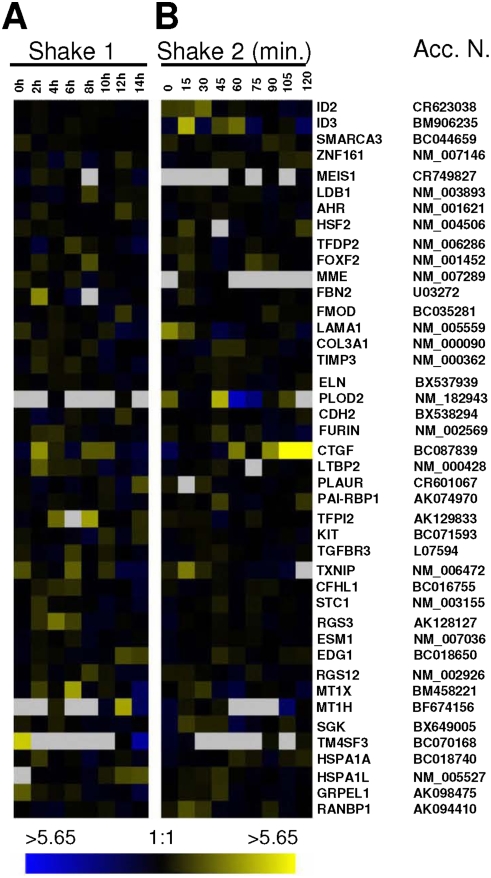
Expression profile of serum-response genes. The data from array experiments (A) *shake 1* and (B) *shake 2* were analyzed as in Fig. 2 for genes identified in serum-response experiments [7].

**Figure 5:**
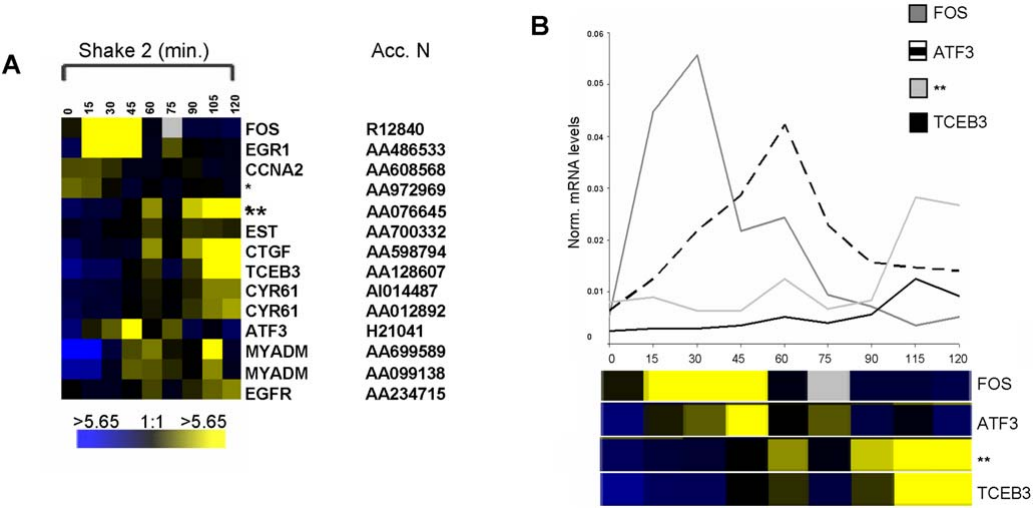
Gene activity during the first 2 hours of G1 phase in HeLa cells. (A) Expression profile of selected G1-regulated genes out of the list of 200 (see supplementary Table S1). The data shown here is derived from mRNAs collected at 15 min intervals over the first 2 hours after plating, in early G1. The analysis performed here is as in Fig. 2. For more information on how these genes were identified, refer to the methods section. (B) Quantitative RT-PCR analysis of candidate RNAs in synchronously growing cells in early G1 by real-time amplification (BioRad). The graph generated represents normalized mRNA levels of each gene under study relative to actin and the bar below each graph outlines the array expression patterns of candidate genes shown in (A). The values on the Y-axis are arbitrary and are the results of normalizing to α-actin RNA levels in the same sample. Refer to (A) for comparison – yellow represents *high* and blue *low* level of expression. Gene symbols are shown, (*) (**) *hypothetical protein*, (Acc. N.) *accession number*.

### No Induction of Mechanical Stress Genes by Mitotic Selection

Since our goal was to examine gene activity in early G1 of normal, unperturbed cycling cells, it was important to inspect the activity of genes which might be activated by stress, in particular, mechanical stress, in cells obtained by mitotic shake-off. In the analyses presented in Figure 3, we examined the activity of genes identified by other investigators in the scientific literature as stress response genes, which might be activated by mitotic shake-off. We analyzed those genes identified as activated by shear force [30], cyclic pressure [31], hydrostatic pressure [32] and mechanical stress [33]. These genes include NF-kB (nuclear factor kB), *jun*, *sod3* (superoxide dismutase 3) and *cycs* (cytochrome c). Figure 3A (*shake 1*) and 3B (*shake 2*) profile the relative change in gene expression of these stress genes [30]–[33] across the 14 hour time period of *shake 1* and the first two hours of G1 phase (*shake 2*). None of these mechanical stress genes show significant upregulation in gene expression by mitotic selection. These data indicate that the mitotic selection method is a stress-free system as it does not activate the mechanically-induced genes. *Shake 2* results are particularly important, since these gene arrays profile gene activity every 15 minutes from collection of late telophase cells by shake-off through the first two hours of G1 phase.

### No Activation of Serum-Response Genes by Mitotic Selection

To validate the comparison of our gene profiles for early G1 phase to the many studies involving the restriction point later in G1, we examined the set of genes identified as being activated in response to serum stimulation [7]. Figure 4(A,B) shows the relative activity of those genes identified in the previous microarray studies of the effect of the serum response in our two experimental sets of RNA. All of the genes identified as activated in the serum response, with the exception of CTGF, show no significant upregulation in gene expression in our datasets. The analysis of genes identified as stress-response genes in the serum starvation experiments [7] shows clearly that these genes are not activated by mitotic shake-off. This demonstrates that the mitotic shake-off technique is a reliable method of achieving cell synchrony without activating stress response genes. This method allows for examination of cell cycle events in cells progressing normally into a new cell cycle.

### Identification of Genes Up- or Down-Regulated in Early G1

Our analysis of genome activity over the first two hours of a new cell cycle (*shake 2*) was based upon 9 gene arrays. Total RNA was isolated every 15 minutes from 0 time (late telophase) to 2 hours after mitotic selection. RNAs from populations harvested at these time points were hybridized to slides containing 29,000 gene targets. Our goal for this study was to identify those genes whose activity was the most highly variable over the two hour period of early G1.

Our statistical analyses of this large dataset (9 slides×43,000 cDNAs) identified the two hundred genes whose activity was the most variable over the two hour period examined. This list is available as a supplementary data (supplementary Table S1). The genes on the list are rank-ordered in terms of relative variability, with *fos* showing the highest variability over the first two hours of G1 phase of any of the 29,000 genes on the arrays. Conversely, *cyr61* is 193 and 196 in the rank order. Comparison of the two different *cyr61* cDNAs on different parts of the slide array which hybridized to the same RNA is an example of the intentional redundancy of these arrays and serves as internal control for the hybridizations. The list of 200 genes contains known cell cycle and other growth regulatory genes, as well as many others whose products and functions are unknown.

A small number of these genes with highly variable expression in the first 2 hours of G1 phase were selected from supplementary Table S1 and are presented in Figure 5A as gene expression profiles based upon intensities relative to the reference set of RNAs (from asynchronous HeLa cells). In this group, *c-fos*, *egr1 and ccna2* gene products are known growth regulators. Transcription factors, c-Fos and the early growth response protein 1, show up-regulation in early G1 in our study and have variously been shown to play roles in cell growth, proliferation, differentiation and tumorigenesis [34]–[36]. Cyclin A2, showing down-regulation in the earliest time points of G1, is important in S phase regulation, in complex with a cdk, and is active until mitosis [18]. Cyr61 is upregulated in our study and is a product of a growth factor-inducible immediate early response gene which promotes proliferation and adhesion [37]. This gene was recently shown to be one of the most variable genes in a study of the initial 12 hours of mouse embryonic stem cell variation [38]. ATF3 is also a transcription factor with known activities in proliferation as well as in physiological stress response [39] and is up-regulated at the mid-point of our experiment. EGFR, epidermal growth factor receptor, is involved in signaling events in cell proliferation and cancer [40]. CTGF is a growth factor (connective tissue growth factor) involved in signaling events involved in cell growth and maintenance. MYADM, myeloid-associated differentiation marker is a membrane protein implicated in differentiation and cancer [41]. TCEB3, transcription elongation factor B (SIII), polypeptide 3 (110 kDa), is part of the protein complex which activates elongation by RNA polymerase II [42]. The remaining two genes, whose activity is profiled in Figure 5A, hybridized to cDNAs identified as hypothetical proteins or EST and are unknown, as are 47% of the genes on the list of 200.

Verification of the variable gene expression we observed in the array experiment *shake 2* was obtained for several genes by real-time quantitative RT-PCR. Our goal was to examine the overall pattern of mRNA levels normalized to a constitutively expressed gene (actin), which was shown to not vary in previous studies [5]. Figure 5B displays the data from amplified RNA generated using total RNA from *shake 2*. Similar results were observed when using total RNA with no amplification step (data not shown). Specific primers for *c-fos*, *atf3*, *tceb3* and *a hypothetical protein* were used to quantitate the relative amounts of RNA in the 9 experimental samples from *shake 2*. The results are presented graphically. The profile of gene activity for these genes is directly comparable to that observed in Figure 5A, (compare profile below each graph) validating the results of our analysis (supplementary Table S1).

In addition, Tables S2 and S3 contain supplementary data which list the 100 genes from our genome-scale analysis which have the highest relative gene activity on any of the 9 experimental array slides (Table S2), or the 100 genes in the human genome with the lowest relative gene activity at any time point in *shake 2* (Table S3). Not surprisingly, some of the genes in the list of 200, whose relative expression is the most variable over first two hours of G1, are on Tables S2 or S3. Among the genes listed in Table S1, Table S2, or Table S3 are known growth regulators, as well as unknown proteins which could be important G1 regulators.

## Discussion

Studies of events occurring after the restriction point, through release of cells from growth factor limitation, are the source of much of our knowledge about regulatory proteins in G1 phase of the cell cycle (for a review see reference 14). Knowledge of events occurring after mitosis and before the R point in mammalian cells has come primarily from cells blocked, then released, in S phase or at G2/M by inhibitory drugs [8]–[12].

We used a well-characterized human cancer cell line in which Rb and p53 are inactivated, but genes involved in cell cycle progression, DNA replication, chromosome segregation and cell adhesion show appropriate activity in the cell cycle [5], [43]. Our goal in the studies reported here was to obtain a synchronous population of naturally cycling cells with which to examine gene activity of the human genome in early G1 of the cell cycle. We attempted to establish other human cell lines for these shake-off experiments, including U2OS cells, but were unsuccessful. The success of these experiments depends upon the ability to collect a synchronous population of cells, all in late telophase of mitosis. For human cells growing attached to a growth substrate, early events include adhesion to the growth substrate, production and activation of G1 cyclins, and preparation for entry into S phase of a new cell cycle. Intuitively, it is obvious that growth regulators of the earliest moments of a new cell cycle may be important regulators of cell growth and proliferation in normal as well as tumor cells. Therefore, we used microarray technology to identify the human genes whose activity is the most highly varied in G1 of the cell cycle with the hope that this group of genes may contain future targets for drug development and treatment of human disease.

Methods which involve limitation of growth factors and use of drugs alter cellular events upon re-entry into the cell cycle once the block is removed. For example, cells synchronized using aphidicolin or mimosine show highly elevated levels of checkpoint regulatory proteins, p53 and p21, and these elevated protein levels persist even after the cells are released from the block [44]. Also, cells synchronized by thymidine and/or hydroxyurea show higher expression levels of cyclins A and B1 in S and G2 phases than in mitotically selected cells progressing normally through the cell cycle [45]. The downstream effects of the elevated levels of important regulatory proteins on cellular signal pathways are likely to be significant. And as others have shown, entry into the cell cycle after serum starvation involves the wound healing response, which greatly complicates interpretation of the changes in gene activity as cells progress out of G0 into late G1 [7].

In our studies presented here, we used current traditional methods to show cell synchrony and normal cell cycle progression: BrdU uptake by S phase cells, and FACS. In addition, we examined the datasets generated from our genome-wide study of gene expression in HeLa cells obtained by mitotic selection. These microarray studies involved more than 29,000 human genes arrayed on glass slides which contained a total of 43,000 targets [5]. In these studies, we examined three particular sets of genes to study synchrony as well as stress gene activity in mitotically selected cell populations. First, to examine synchrony of the cell populations, we examined replication-dependent and replication-independent histone gene activity in the two experimental sets of gene arrays. Replication-dependent histone gene expression is tightly regulated in the cell cycle. This family of genes is up-regulated at the G1/S boundary, and down-regulated in mid S-phase [46]. Figure 2 shows the gene expression profile of the replication-dependent genes in a time-series experiment (*shake 1*) that spans a period of 14 hours after plating mitotic cells. The dataset shows that the upregulation of these replication-dependent genes occurs 6 hours after plating, which coincides with G1/S transition in HeLa cells [4], at the time the cell commits to S phase. The replication-dependent histone genes whose activity is shown in Figure 2 represent all the classes of histone proteins present in the nucleosome core complex. The replication-independent histone genes are not up-regulated at G1/S phase transition, but are expressed at relatively low and constitutive levels throughout the cycle [47], [48]. In the second time-series array experiment (*shake 2*), we show that neither replication-dependent or -independent histone genes present on the human gene arrays vary in activity over the first 2 hours of G1 (Figure 2B), verifying both synchrony and expected temporal patterns of histone gene activity.

Next, we examined stress-response gene activity among those known genes which could be considered pertinent to our selection method, gene products associated with mechanical stress. Researchers have identified genes activated under various types of mechanical stress [31]–[33], [44]. Examination of the stress-response gene datasets from the array experiments clearly shows there is no association between stress-response gene activity and the mitotic shake-off method, either in the early time period (2 hours) following selection (*shake 2*) or throughout the first 14 hours of a new cell cycle (*shake 1*). We then examined a set of well-characterized genes that were identified as being activated in serum starved cells, in response to serum stimulation [7]. With the exception of CTGF, this gene set did not show any significant increase in gene activity in our experiments (Fig. 4A, B). We suggest that CTGF upregulation is probably not indicative of cellular stress activity. CTGF is a growth factor and one possible explanation to its upregulation late in early G1 (*shake 2*) is likely due to its involvement in signaling pathways essential for cells to progress in the cell cycle, and particularly beyond G1. The exact role of CTGF in the cell cycle is not completely understood. One study showed that use of anti-sense approach against CTGF reversed angiotensin II-induced renal hypertrophy by releasing renal cells from angiotensin II-induced G0–G1 arrest [49]. In contrast, CTGF was shown to be a mediator of diabetic nephropathy by inducing re-entry into G1 phase [50]. Further, CTGF is an important regulator of fibroblast proliferation in connective tissue in late G1 and into S phase [51]. Clearly the function of CTGF is dependent on cell type or disease state, and our microarray data in unperturbed synchronized cells shows that further experiments are needed to elucidate the function of CTGF in G1 and whether it is required for progression into a new cell cycle.

In contrast to the histone genes, the stress response genes, and genes activated in starved cells upon serum addition to the growth medium, a set of genes was identified as having high variation in activity in early G1 of the cell cycle. We have identified the 200 most highly variable genes among the 29,000 genes represented on the arrays, showing up- or down-regulation over the 2 hours of early G1 phase (see Table S1). Many genes identified here are known to be involved in proliferation and/or cell cycle regulation (cyclin A2 [52], epidermal growth factor receptor [40], early growth response 1 [34], [35] and fos [36]) and show variation in gene activity in expected up- or down- patterns over the time period examined in early G1. However, a number of genes identified in our studies have not been previously characterized and functions of their putative products are not known.

In Figure 5, we showed the gene activity profiles of several known and unknown genes whose activity is highly variable over the two hour time period examined. We verified the accuracy of gene activities produced by hybridization to cDNA arrays for several of these genes by real-time RT-PCR of both total and amplified RNAs, using the original RNA samples from the *shake 2* experiment. Taken together with the gene activity profiles of the families of genes which serve to validate both the temporal window of the cell cycle we examined and the stress-free method by which we synchronized cells, the G1-regulated genes identified here should provide new information and understanding of important events occurring early in the cell cycle.

In summary, we have shown here that the mitotic shake-off technique is a reliable method to study cell cycle events, particularly those occurring early in a new cell cycle. As we have demonstrated, this is a relatively stress-free system. The limitation of the use of this approach is that many cell types, those which attach too tightly to the growth substrate, or alternatively, those which do not shake off in a predictable fashion due to lack of adhesion to the growth surface, can not be synchronized by this technique. Our selection method spans a window of 10 minutes, and ensures that most of the selected cells enter S phase within a relatively short period where over 95% of the selected cells reenter the cell cycle synchronously. We have included data obtained by a variety of experimental methods to show synchrony of the cell populations, ranging from genome-wide scale microarray analyses to FACS analysis of DNA content of the cell populations, proving the merit of the technique. We have also identified a number of potential G1 regulators which may play essential roles in cell cycle progression, normal growth and proliferation, as well as tumorigenesis. We are currently using mitotic selection to obtain cells for the examination of subcellular localization of interesting proteins, and are searching for new regulators of the cell cycle, primarily those essential for progression into and beyond the G1 phase of the human cell division cycle.

## Materials and Methods

### Cell culture, mitotic selection and FACS analysis

Prior to the time of the mitotic selection experiment, cells are grown to the appropriate density in a humidified chamber at 37°C, 5% CO_2_. At the time of the experiment, the flasks are removed from the growth incubator and placed inside the temperature-regulated chamber (37°C), on the vibration platform. A motor under the control of a computer chip vibrates the platform for 15 seconds at 10-minute intervals. After each vibration, cells detached from the flasks were collected and stored on ice. Thus, we select cells detaching from substrate during the previous 10 minutes. The intensity/force of shaking can be adjusted based upon the cell type. HeLa or CHO cells were cultured in 75 cm^2^ flasks. HeLa cells were grown in Dulbecco's Modified Eagle Medium, 10% fetal bovine serum and 1% MEM non-essential amino acids (Sigma M7145). CHO cells were grown in McCoy's 5A medium supplemented with 10% calf serum. Penicillin-streptomycin (Gibco-BRL) at 1% is added to the culture media. For FACS analysis, the cells were harvested at indicated times after mitosis. Cells were stained with propidium iodide as described by others [53]. Briefly, cells were trypsinized, washed twice in PBS, followed by fixation in 70% ethanol on ice for a minimum of 2 hours. Cells were washed 2 additional times in PBS, and then stained for 30 min at 37°C in 50 µg/ml PI solution containing 200 µg/ml RNase A and 0.1% Triton-X-100. Samples were stored at 4°C until analysis on BD Biosciences FACSCanto in the Flow Cytometry core lab at the College of Medicine. A minimum of 10,000 cells was counted and analyzed with BD Biosciences FACSDiva Software.

### BrdU incorporation

HeLa cells were cultured as described above. For bromodeoxyuridine (BrdU) incorporation, the cells were pulse-labeled with the BrdU agent (BrdU labeling and detection kit, Roche Molecular Biochemicals/Boehringer Mannheim) for 30 min before harvest at the designated time post-mitotically. Cells were fixed with 70% ethanol in glycine (15 mM, pH 2.0) for 20–30 min at −20°C, and then washed 3 times with PBS. Fixed cells were prepared for immunocytochemistry as described earlier [4], [54]. Briefly, cells were incubated with primary antibodies for detection of the BrdU agent, and then washed for 10 min with PBS. Secondary antibody (from BrdU kit) incubation was for 40 min. Cells were washed again and counterstained with DAPI at room temperature for 10 min and mounted in Vectashield (Vector Laboratories). For Figure 1A, images were captured with an Olympus IMT-2, Delta Vision (Applied Precision) deconvolution microscope with a 60× objective lens. For Figure 1B, images were captured using QFM inverted microscope.

### Microarray analysis

Mitotic cells were collected using mitotic shake-off. Total RNA was prepared using ULTRASPEC RNA isolation system (Biotecx, Houston, TX). Reference RNA was prepared from asynchronously growing HeLa cells using TRIzol (Invitrogen). For cDNA synthesis and microarray hybridization, refer to Whitfield et al. [5]. Herein, we used the Stanford Microarray Database (SMD) (http://genome-www5.stanford.edu//) to analyze, sort and cluster microarray raw data. For data retrieval, the normalized ratio of mean intensities from the experimental and control samples was considered. The genes selected for analysis were chosen using their corresponding clone IDs. Gene names and accession numbers displayed in all figures were generated from the SMD online analysis software, and accession numbers were further verified using the S.O.U.R.C.E online tool (http://genome-www5.stanford.edu/cgi-bin/source/sourceSearch). Two shake-off experiments were used in these analyses, *shake 1*, which was conducted over 14 hours and samples collected every 2 hrs, and *shake 2* which was conducted over 2 hours and samples collected every 15 minutes. A previous paper [5] utilized RNA samples from *shake 1*, from double-thymidine blocked cells, and from thymidine-nocodozole blocked cells and compared data from all three synchronization methods to identify cell cycle regulated genes common to all three experimental sets. Here we analyze changes in gene expression over the course of *shake 1* and *shake 2* to identify G1-regulated genes, producing data sets unique to this analysis. The data discussed in this manuscript have been deposited in NCBI's Gene Expression Omnibus [55], [56] and are accessible through GEO Series accession number GSE12473 (http://www.ncbi.nlm.nih.gov/geo/query/acc.cgi?accGSE12473)

### Gene Ontology (GO) Analysis

UniGene mRNA accession numbers, representative of identified genes, were generated from Clone IDs using SOURCE (http://source.stanford.edu), and Gene Ontology analysis was conducted using the Database for Annotation, Visualization, and Integrated Discovery (DAVID) [57]. Briefly, SOURCE-generated list was uploaded and subsequently converted to DAVID IDs using the Gene Functional Classification tool (http://david.abcc.ncifcrf.gov/gene2gene.jsp). GO biological process annotations were analyzed using the Functional Annotation Tool. GO biological process terms that were at least 2-fold enriched and had a false discovery rate of less than 10% are shown in Table S4.

### G1-regulated Genes and Statistical Analyses

Our interest was not in the cyclic expression of genes during the cell cycle, but rather examining the variability of expression. To discover the genes presenting the most variable expression we used as our basic measure the logarithm (base 2) of ratio of experimental to control normalized intensities. We calculated the standard deviation of the log2 ratio of these intensities amongst the nine times (0, 15, 30, 45, 60, 75, 90, 105, and 120 minutes) at which expression was determined for each spot on the array. We then ordered these standard deviations and focused on those genes with the highest variability.

### Real Time RT-PCR

Microarray data was confirmed using either total or amplified RNAs from original samples. Total RNAs were amplified using MessageAmp™ aRNA Kit (Ambion) per instructions of the manufacturer. RNA samples were quantified by UV absorbance spectrophotometry at 260 nm, or NanoDrop® (ND-1000 spectrophotometer) and were stored in RNase-free water at −70°C. The aRNAs were run on 1.25% agarose gel to check integrity and correct size of the synthesized aRNA products as determined by ethidium bromide staining. For reverse transcriptase (RT) - reaction, equal amounts (250 ng) of total RNA or 1 µg of aRNA from each sample were used. First strand cDNA synthesis was done in 20-µl reaction containing a random hexamer, dNTPs at 10 mM, and RT enzyme (New England Biolabs) at 15 units and standard buffer. The cycle was 65°C for 5 minutes, ice (2–5 minutes), 25°C for 10 minutes, 37°C for 45 minutes, 85°C for 5 minutes and 4°C for 10 minutes. One-tenth (2 µl) of the cDNA reaction volume was amplified in 25-µl reactions containing the SYBR Green mix and each primer set designed to amplify specifically the transcribed region of the candidate genes, or α-actin (see below) using the BioRad iCycler iQ real-time PCR detection system. The cycles were 95°C for 5 minutes, (95°C for 15 seconds, 55°C for 30 seconds, 72°C for 30 seconds) repeated 40 times, 95°C for 1 minute and 55°C for 1 minute, followed by a cycle of an increment increase of 0.4°C repeated 100 times for melt curve data collection and analysis. α-actin primers were used to verify changes in experimental RNAs across the time points and α-actin was chosen because it is not regulated in the HeLa cell cycle [5]. To rule out contamination, all amplified reactions were run on 1.25% agarose gel to ensure the presence of one unique product and running at the appropriate molecular weight DNA marker. Primer sequences are as follows:

*fos* (forward primer) 5′ AGA TTG CCA ACC TGC TGA AGG AGA 3′;
*fos* (reverse primer) 5′ TGG ATG ATG CTG GGA ACA GGA AGT 3′;
*tceb* (forward primer) 5′ AGA AAT CAC ACA AGG CCC TCT CCA 3′;
*tceb* (reverse primer) 5′ TTT ACC TTG GGC AAC AGG TCT CCT 3′;
*hypothetical protein* (forward primer) 5′ TCG TAT GCA GAA TCT GTG GGA GCA 3′;
*hypothetical protein* (reverse primer) 5′ TGG TCT GGG CTT GAG GTT CAT CAT 3′;
*atf3* (forward primer) 5′ TCA AGG AAG AGC TGA GGT TTG CCA 3′;
*atf3* (reverse primer) 5′ CTT CTT GTT TCG GCA CTT TGC AGC 3′;
α-*actin* (forward primer) 5′ GTG CGT GAC ATT AAG GAG AAG 3′;
α-*actin* (reverse primer) 5′ GAA GGT AGT TTC GTG GAT GCC 3′.

## Supporting Information

Table S1 List of 200 highly variable genes (Shake 2). Genome-scale analysis of G1-regulated genes. The identified genes are presented using their corresponding clone IDs. Gene names and accession numbers displayed in all tables were generated from the SMD online analysis software (http://genome-www5.stanford.edu/), and accession numbers were further verified using the S.O.U.R.C.E online tool (http://genome-www5.stanford.edu/cgi-bin/source/sourceSearch). The full data is available online (http://www.ncbi.nlm.nih.gov/geo/query/acc.cgi?accGSE12473)
(0.25 MB DOC)

Table S2 List of 100 genes with highest expression at any time point (Shake 2). Genome-scale analysis of G1-regulated genes. The identified genes are presented using their corresponding clone IDs. Gene names and accession numbers displayed in all tables were generated from the SMD online analysis software (http://genome-www5.stanford.edu/), and accession numbers were further verified using the S.O.U.R.C.E online tool (http://genome-www5.stanford.edu/cgi-bin/source/sourceSearch). The full data is available online (http://www.ncbi.nlm.nih.gov/geo/query/acc.cgi?accGSE12473)
(0.13 MB DOC)

Table S3 List of 100 genes with lowest expression at any time point (Shake 2). Genome-scale analysis of G1-regulated genes. The identified genes are presented using their corresponding clone IDs. Gene names and accession numbers displayed in all tables were generated from the SMD online analysis software (http://genome-www5.stanford.edu/), and accession numbers were further verified using the S.O.U.R.C.E online tool (http://genome-www5.stanford.edu/cgi-bin/source/sourceSearch). The full data is available online (http://www.ncbi.nlm.nih.gov/geo/query/acc.cgi?accGSE12473)
(0.14 MB DOC)

Table S4 GO analysis. Gene ontology analysis of G1 genes (see supplemental Table S1). Columns represent GO “biological process” category, number of genes under that category (# of Genes), representation in percent (%) to total number of genes (127, output of DAVID analysis), p-value, fold enrichment and false discovery rate (FDR<10). For further information see methods section.
(0.05 MB DOC)
