# Supplementary material for: Identification of G1-Regulated Genes in Normally Cycling Human Cells
Source: PLoS One. 2008 Dec 15;3(12):e3943. doi: 10.1371/journal.pone.0003943 (PMC2600614; doi:10.1371/journal.pone.0003943)
Supplement: Table S1 — List of 200 highly variable genes (Shake 2). Genome-scale analysis of G1-regulated genes. The identified genes are presented using their corresponding clone IDs. Gene names and accession numbers displayed in all tables were generated from the SMD online analysis software (http://genome-www5.stanford.edu/), and accession numbers were further verified using the S.O.U.R.C.E online tool (http://genome-www5.stanford.edu/cgi-bin/source/sourceSearch). The full data is available online (http://www.ncbi.nlm.nih.gov/geo/query/acc.cgi?accGSE12473) (0.25 MB DOC) [file pone.0003943.s001.doc]

| **#** | **Cloneid** | **Genesymbol** | **Genename** | **Acc. Num.** |
| --- | --- | --- | --- | --- |
| 1 | IMAGE:26474 | FOS | v-fos FBJ murine osteosarcoma viral oncogene homolog | NP_005243 |
| 2 | IMAGE:1741473 |  | ESTs |  |
| 3 | IMAGE:357396 | MUC15 | mucin 15 | NP_663625 |
| 4 | IMAGE:452545 |  | ESTs |  |
| 5 | IMAGE:1732822 |  | ESTs |  |
| 6 | IMAGE:1737622 |  | ESTs, Highly similar to plakophilin 2 [H.sapiens] |  |
| 7 | 1292741 |  | EST |  |
| 8 | IMAGE:454981 |  | ESTs |  |
| 9 | IMAGE:526184 | RAB21 | RAB21, member RAS oncogene family |  |
| 10 | IMAGE:898092 | CTGF | connective tissue growth factor | NP_001892 |
| 11 | IMAGE:526657 | TCEB3 | transcription elongation factor B (SIII), polypeptide 3 (110kDa, elongin A) | NP_003189 |
| 12 | IMAGE:49131 |  | ESTs |  |
| 13 | IMAGE:450043 | PSG6 | pregnancy specific beta-1-glycoprotein 6 |  |
| 14 | IMAGE:109123 |  | H. sapiens cDNA FLJ36837 fis, clone ASTRO2011422, mRNA sequence |  |
| 15 | IMAGE:230613 |  | EST |  |
| 16 | IMAGE:150216 | KIAA1337 | KIAA1337 protein |  |
| 17 | IMAGE:300973 | CD8A | CD8 antigen, alpha polypeptide (p32) | NP_741969 |
| 18 | IMAGE:435970 |  | ESTs |  |
| 19 | IMAGE:1736819 | UACA | uveal autoantigen with coiled-coil domains and ankyrin repeats | NP_060473 |
| 20 | IMAGE:545242 | STAT1 | signal transducer and activator of transcription 1, 91kDa | NP_644671 |
| 21 | IMAGE:811048 | PLXND1 | Plexin D1 | NP_055918 |
| 22 | 1293010 |  | ESTs |  |
| 23 | IMAGE:815816 | VDR | vitamin D (1,25- dihydroxyvitamin D3) receptor | NP_001017535 |
| 24 | IMAGE:1891471 |  | ESTs |  |
| 25 | IMAGE:277305 | RHOB | Ras homolog gene family, member B | NP_004031 |
| 26 | IMAGE:1508768 | TMEM16B | Transmembrane protein 16B | NP_065106 |
| 27 | IMAGE:1911721 | GNAS | GNAS complex locus | NP_536351 |
| 28 | IMAGE:1010523 | SCUBE2 | **signal peptide, CUB domain, EGF-like 2 |  |
| 29 | IMAGE:754149 |  | IQGAP1 protein [Homo sapiens], mRNA sequence | NP_003861 |
| 30 | IMAGE:759163 | MFAP4 | microfibrillar-associated protein 4 | NP_002395 |
| 31 | IMAGE:292612 | SUCLG2 | **succinate-CoA ligase, GDP-forming, beta subunit |  |
| 32 | IMAGE:377433 | BBS7 | Bardet-Biedl syndrome 7 | NP_789794 |
| 33 | IMAGE:814218 |  | ESTs |  |
| 34 | IMAGE:859228 | IDH1 | isocitrate dehydrogenase 1 (NADP+), soluble |  |
| 35 | IMAGE:840944 | EGR1 | early growth response 1 |  |
| 36 | IMAGE:253009 | MALAT1 | Metastasis associated lung adenocarcinoma transcript 1 |  |
| 37 | IMAGE:1500387 | EPC1 | enhancer of polycomb homolog 1, (Drosophila) | NP_079485 |
| 38 | IMAGE:503155 | AP4E1 | Adaptor-related protein complex 4, epsilon 1 subunit | NP_031373 |
| 39 | IMAGE:1475120 | CUL5 | Cullin 5 | NP_003469 |
| 40 | IMAGE:1253352 | CSH2 | chorionic somatomammotropin hormone 2 |  |
| 41 | IMAGE:1891605 | MMP28 | Matrix metallopeptidase 28 | NP_116568 |
| 42 | IMAGE:79692 | SFTPA2 | surfactant, pulmonary-associated protein A2 | NP_008857 |
| 43 | IMAGE:811953 | MCM4 | **MCM4 minichromosome maintenance deficient 4 (S. cerevisiae) |  |
| 44 | IMAGE:767469 | ANKH | **ankylosis, progressive homolog (mouse) |  |
| 45 | IMAGE:795288 | USP4 | Ubiquitin specific peptidase 4 (proto-oncogene) | NP_955475 |
| 46 | IMAGE:433491 | MYADM | Myeloid-associated differentiation marker | NP_612382 |
| 47 | IMAGE:1668067 | DKFZP434D146 | DKFZP434D146 protein |  |
| 48 | IMAGE:290263 |  | ESTs |  |
| 49 | IMAGE:364685 | CENTB5 | centaurin, beta 5 | NP_085152 |
| 50 | IMAGE:1204078 |  | EST |  |
| 51 | IMAGE:1526609 | PIGN | Phosphatidylinositol glycan anchor biosynthesis, class N | NP_789744 |
| 52 | IMAGE:147834 | ZNF217 | zinc finger protein 217 | NP_006517 |
| 53 | IMAGE:41029 | CLASP1 | cytoplasmic linker associated protein 1 | NP_056097 |
| 54 | IMAGE:219898 | LIMCH1 | LIM and calponin homology domains 1 | NP_055803 |
| 55 | IMAGE:488010 | LIMCH1 | LIM and calponin homology domains 1 |  |
| 56 | IMAGE:949944 | CASP2 | caspase 2, apoptosis-related cysteine protease (neural precursor cell expressed, developmentally down-regulated 2) | NP_116766 |
| 57 | IMAGE:1684695 | CCDC132 | Coiled-coil domain containing 132 | NP_078829 |
| 58 | IMAGE:1631116 |  | ESTs |  |
| 59 | IMAGE:343978 |  | EST |  |
| 60 | IMAGE:296141 | KBTBD2 | Kelch repeat and BTB (POZ) domain containing 2 | NP_056298 |
| 61 | IMAGE:745100 |  | ESTs |  |
| 62 | IMAGE:211832 |  | ESTs |  |
| 63 | IMAGE:259066 | FNDC3B | Fibronectin type III domain containing 3B | NP_073600 |
| 64 | IMAGE:1696157 |  | H. sapiens mRNA; cDNA DKFZp762N156, mRNA sequence |  |
| 65 | IMAGE:35626 | SLC38A1 | Solute carrier family 38, member 1 | NP_109599 |
| 66 | IMAGE:377193 | DHX29 | DEAH (Asp-Glu-Ala-His) box polypeptide 29 | NP_061903 |
| 67 | IMAGE:826350 | GPS1 | G protein pathway suppressor 1 | NP_997657 |
| 68 | IMAGE:364209 | ANK3 | ankyrin 3, node of Ranvier (ankyrin G) |  |
| 69 | IMAGE:1876405 |  | Homo sapiens partial mRNA; ID EE2-8E, mRNA sequence |  |
| 70 | IMAGE:455136 |  | ESTs |  |
| 71 | IMAGE:239792 |  | ESTs |  |
| 72 | IMAGE:148740 |  | Hypothetical protein [Homo sapiens], mRNA sequence |  |
| 73 | IMAGE:784847 | CTR9 | Ctr9, Paf1/RNA polymerase II complex component, homolog (S. cerevisiae) | NP_055448 |
| 74 | IMAGE:1043332 |  | ESTs |  |
| 75 | IMAGE:753760 |  | EST |  |
| 76 | IMAGE:1416166 |  | ESTs |  |
| 77 | IMAGE:626531 | NSAP1 | NS1-associated protein 1 |  |
| 78 | IMAGE:1732539 |  | EST |  |
| 79 | IMAGE:200855 |  | ESTs, Weakly similar to hypothetical protein FLJ20378 [H.sapiens] |  |
| 80 | IMAGE:79231 |  | EST |  |
| 81 | IMAGE:1190676 |  | EST |  |
| 82 | IMAGE:754541 | ZMYM2 | Zinc finger, MYM-type 2 | NP_932072 |
| 83 | IMAGE:204755 | SFRS11 | splicing factor, arginine/serine-rich 11 | NP_004759 |
| 84 | IMAGE:1902500 |  | Unnamed protein product [Homo sapiens], mRNA sequence |  |
| 85 | IMAGE:462544 | MAT1A | methionine adenosyltransferase I, alpha | NP_000420 |
| 86 | IMAGE:1859075 |  | EST |  |
| 87 | IMAGE:143790 | OGT | O-linked N-acetylglucosamine (GlcNAc) transferase (UDP-N-acetylglucosamine:polypeptide-N-acetylglucosaminyl transferase) | NP_858059 |
| 88 | IMAGE:1674087 | SLC35E1 | Solute carrier family 35, member E1 | NP_079157 |
| 89 | IMAGE:1509553 |  | ESTs |  |
| 90 | IMAGE:220376 | TncRNA | Trophoblast-derived noncoding RNA |  |
| 91 | IMAGE:669485 | EGFR | epidermal growth factor receptor (erythroblastic leukemia viral (v-erb-b) oncogene homolog, avian) | NP_958441 |
| 92 | IMAGE:897102 | TXNDC10 | Thioredoxin domain containing 10 | NP_061895 |
| 93 | IMAGE:1672662 |  | ESTs |  |
| 94 | IMAGE:1896495 | SOX1 | SRY (sex determining region Y)-box 1 | NP_005977 |
| 95 | IMAGE:1505470 | PRO2133 | hypothetical protein PRO2133 |  |
| 96 | IMAGE:812266 | MAN1A2 | mannosidase, alpha, class 1A, member 2 | NP_006690 |
| 97 | IMAGE:1535755 |  | ESTs |  |
| 98 | IMAGE:950381 | RPL18A | **ribosomal protein L18a |  |
| 99 | IMAGE:1011159 | AHI1 | Abelson helper integration site 1 | NP_060121 |
| 100 | IMAGE:1626269 |  | ESTs |  |
| 101 | IMAGE:198011 |  | ESTs |  |
| 102 | IMAGE:147133 |  | ESTs |  |
| 103 | IMAGE:1628456 | ANKRD11 | Ankyrin repeat domain 11 | NP_037407 |
| 104 | IMAGE:712347 | VCPIP1 | Valosin containing protein (p97)/p47 complex interacting protein 1 | NP_079330 |
| 105 | IMAGE:1473257 | TM4SF18 | Transmembrane 4 L six family member 18 | NP_620141 |
| 106 | IMAGE:664968 | MGA | MAX gene associated |  |
| 107 | IMAGE:281103 | ASH1 | hypothetical protein ASH1 | NP_060959 |
| 108 | IMAGE:782209 | BBX | bobby sox homolog (Drosophila) | NP_064620 |
| 109 | IMAGE:1475726 | C14orf83 | chromosome 14 open reading frame 83 | NP_872332 |
| 110 | IMAGE:487831 |  | H. sapiens cDNA FLJ14059 fis, clone HEMBB1000573, mRNA seq. |  |
| 111 | IMAGE:85224 | RBM25 | RNA binding motif protein 25 | NP_067062 |
| 112 | IMAGE:247783 | SNAP29 | synaptosomal-associated protein, 29kDa | NP_004773 |
| 113 | IMAGE:950459 |  | H.sapiens mRNA (fetal brain cDNA h6_2g), mRNA sequence |  |
| 114 | IMAGE:1535554 | BTC | betacellulin | NP_001720 |
| 115 | IMAGE:504544 | HCK | hemopoietic cell kinase | NP_002101 |
| 116 | IMAGE:460590 |  | ESTs |  |
| 117 | IMAGE:346930 |  | EST |  |
| 118 | IMAGE:1948395 | ASXL1 | Additional sex combs like 1 (Drosophila) | NP_056153 |
| 119 | IMAGE:746303 |  | ESTs |  |
| 120 | IMAGE:1578408 | MGC29937 | hypothetical protein MGC29937 |  |
| 121 | IMAGE:460665 |  | ESTs |  |
| 122 | IMAGE:307553 | KRAS2 | v-Ki-ras2 Kirsten rat sarcoma 2 viral oncogene homolog | NP_203524 |
| 123 | IMAGE:1841678 |  | ESTs |  |
| 124 | IMAGE:142087 |  | ESTs |  |
| 125 | IMAGE:800137 | PAX2 | paired box gene 2 | NP_003981 |
| 126 | IMAGE:294190 | XRN1 | 5'-3' exoribonuclease 1 | NP_061874 |
| 127 | IMAGE:1473682 | ZNF414 | Zinc finger protein 414 | NP_115746 |
| 128 | IMAGE:51448 | ATF3 | activating transcription factor 3 | NP_004015 |
| 129 | IMAGE:46011 | TBC1D24 | TBC1 domain family, member 24 |  |
| 130 | IMAGE:81601 | HLF | **hepatic leukemia factor | NP_002117 |
| 131 | IMAGE:1588477 | CALCRL | calcitonin receptor-like | NP_005786 |
| 132 | IMAGE:489495 | LOC91663 | hypothetical protein BC013995 | NP_612382 |
| 133 | IMAGE:448323 |  | ESTs |  |
| 134 | IMAGE:416744 | CTBP2 | C-terminal binding protein 2 | NP_073713 |
| 135 | 1049000 | BCR | breakpoint cluster region |  |
| 136 | IMAGE:1534890 | ANKRD12 | Ankyrin repeat domain 12 | NP_056023 |
| 137 | IMAGE:1502566 | NMNAT3 | Nicotinamide nucleotide adenylyltransferase 3 | NP_835471 |
| 138 | IMAGE:26505 | DPYSL5 | Dihydropyrimidinase-like 5 | NP_064519 |
| 139 | IMAGE:1901128 | FLJ32069 | hypothetical protein FLJ32069 |  |
| 140 | IMAGE:434167 |  | Homo sapiens cDNA FLJ12308 fis, clone MAMMA1001931, mRNA sequence |  |
| 141 | IMAGE:795820 | SPINK5L3 | Serine PI Kazal type 5-like 3 |  |
| 142 | IMAGE:1745093 | TRIM26 | Tripartite motif-containing 26 | NP_003440 |
| 143 | IMAGE:1543200 |  | ESTs |  |
| 144 | IMAGE:261444 | MYADM | Myeloid-associated differentiation marker | NP_612382 |
| 145 | IMAGE:1655309 |  | ESTs |  |
| 146 | IMAGE:392092 | CDH4 | cadherin 4, type 1, R-cadherin (retinal) | NP_001785 |
| 147 | IMAGE:418340 |  | H. sapiens full length insert cDNA clone ZD45G11, mRNA seq. |  |
| 148 | IMAGE:1738537 | SNTB2 | Syntrophin, beta 2 (dystrophin-associated protein A1, 59kDa, basic component 2) | NP_570896 |
| 149 | IMAGE:344648 | KLF2 | Kruppel-like factor 2 (lung) | NP_057354 |
| 150 | IMAGE:1846815 |  | ESTs |  |
| 151 | IMAGE:713127 | SMC4L1 | SMC4 structural maintenance of chromosomes 4-like 1 (yeast) | NP_005487 |
| 152 | IMAGE:48182 | PGM5 | **phosphoglucomutase 5 |  |
| 153 | IMAGE:194811 |  | H. sapiens full length insert cDNA clone YP91F02, mRNA sequence |  |
| 154 | IMAGE:143896 |  | H. sapiens cDNA FLJ36355 fis, clone THYMU2007384, mRNA seq. |  |
| 155 | IMAGE:1841513 |  | EST |  |
| 156 | IMAGE:1568891 |  | EST |  |
| 157 | IMAGE:1741810 | KIAA0254 | KIAA0254 gene product |  |
| 158 | IMAGE:192419 | VSTM2L | V-set and transmembrane domain containing 2 like | NP_542174 |
| 159 | IMAGE:898333 |  | ESTs |  |
| 160 | IMAGE:171753 |  | Surfactant associated protein F mRNA, partial sequence |  |
| 161 | IMAGE:1256764 | SF3B1 | splicing factor 3b, subunit 1, 155kDa |  |
| 162 | IMAGE:1737593 |  | EST |  |
| 163 | IMAGE:1687719 |  | EST |  |
| 164 | IMAGE:745431 | FAM87B | Family with sequence similarity 87, member B |  |
| 165 | IMAGE:79043 |  | ESTs |  |
| 166 | IMAGE:49524 | QSER1 | Glutamine and serine rich 1 | NP_079050 |
| 167 | IMAGE:1847063 |  | ESTs |  |
| 168 | IMAGE:624271 | CAMKK2 | calcium/calmodulin-dependent protein kinase kinase 2, beta | NP_757380 |
| 169 | IMAGE:824465 | GTF2A1 | general transcription factor IIA, 1, 19/37kDa | NP_963889 |
| 170 | IMAGE:1955637 | PPP1R15B | protein phosphatase 1, regulatory (inhibitor) subunit 15B | NP_116222 |
| 171 | IMAGE:46506 |  | H. sapiens mRNA full length insert cDNA EUROIMAGE 46506, mRNA seq. |  |
| 172 | IMAGE:1500387 | EPC1 | enhancer of polycomb homolog 1, (Drosophila) | NP_079485 |
| 173 | IMAGE:28096 |  | EST |  |
| 174 | IMAGE:451506 |  | ESTs |  |
| 175 | IMAGE:823940 | TOB1 | transducer of ERBB2, 1 | NP_005740 |
| 176 | IMAGE:1738698 | C14orf28 | chromosome 14 open reading frame 28 | NP_001017923 |
| 177 | IMAGE:1841381 | BRWD1 | Bromodomain and WD repeat domain containing 1 | NP_387505 |
| 178 | IMAGE:1741489 | FBXO22 | F-box only protein 22 | NP_671717 |
| 179 | IMAGE:950690 | CCNA2 | cyclin A2 | NP_001228 |
| 180 | IMAGE:1493345 |  | ESTs |  |
| 181 | IMAGE:916796 |  | EST |  |
| 182 | IMAGE:461464 |  | EST | NP_055693 |
| 183 | IMAGE:416567 | SERPINA5 | serine (or cysteine) proteinase inhibitor, clade A (alpha-1 antiproteinase, antitrypsin), member 5 | NP_000615 |
| 184 | IMAGE:129616 |  | ESTs |  |
| 185 | IMAGE:1581817 |  | ESTs |  |
| 186 | IMAGE:1566915 | GRIP1 | Glutamate receptor interacting protein 1 |  |
| 187 | IMAGE:1541376 |  | EST |  |
| 188 | IMAGE:214133 | BRD2 | bromodomain containing 2 | NP_005095 |
| 189 | IMAGE:502351 | AKAP13 | A kinase (PRKA) anchor protein 13 | NP_658913 |
| 190 | IMAGE:294995 | PAFAH1B2 | Platelet-activating factor acetylhydrolase, isoform Ib, beta subunit 30kDa | NP_002563 |
| 191 | IMAGE:1759475 | MAN1C1 | Mannosidase, alpha, class 1C, member 1 | NP_065112 |
| 192 | IMAGE:1525504 |  | ESTs |  |
| 193 | IMAGE:1607286 | CYR61 | cysteine-rich, angiogenic inducer, 61 | NP_001545 |
| 194 | IMAGE:1541256 | FLJ44379 | Similar to S-100 protein, alpha chain |  |
| 195 | IMAGE:1569516 | ASCL1 | achaete-scute complex-like 1 (Drosophila) | NP_004307 |
| 196 | IMAGE:360254 | CYR61 | cysteine-rich, angiogenic inducer, 61 | NP_001545 |
| 197 | IMAGE:916038 |  | EST |  |
| 198 | 1293135 |  | ESTs |  |
| 199 | IMAGE:364541 | RAPGEF2 | Rap guanine nucleotide exchange factor (GEF) 2 |  |
| 200 | IMAGE:1190979 |  | ESTs |  |

Table S1: List of 200 highly variable genes (*Shake 2*)
