# Supplementary material for: Identification of G1-Regulated Genes in Normally Cycling Human Cells
Source: PLoS One. 2008 Dec 15;3(12):e3943. doi: 10.1371/journal.pone.0003943 (PMC2600614; doi:10.1371/journal.pone.0003943)
Supplement: Table S2 — List of 100 genes with highest expression at any time point (Shake 2). Genome-scale analysis of G1-regulated genes. The identified genes are presented using their corresponding clone IDs. Gene names and accession numbers displayed in all tables were generated from the SMD online analysis software (http://genome-www5.stanford.edu/), and accession numbers were further verified using the S.O.U.R.C.E online tool (http://genome-www5.stanford.edu/cgi-bin/source/sourceSearch). The full data is available online (http://www.ncbi.nlm.nih.gov/geo/query/acc.cgi?accGSE12473) (0.13 MB DOC) [file pone.0003943.s002.doc]

| **#** | **Cloneid** | **Gene Symbol** | **Gene Name** | **Acc. Num.** |
| --- | --- | --- | --- | --- |
| 1 | IMAGE:26474 | FOS | V-fos FBJ murine osteosarcoma viral oncogene homolog | BX647104 |
| 2 | IMAGE:357396 | MUC15 | Mucin 15, cell surface associated | AK128337 |
| 3 | IMAGE:840944 | EGR1 | early growth response 1 |  |
| 4 | IMAGE:450043 | PSG6 | pregnancy specific beta-1-glycoprotein 6 |  |
| 5 | IMAGE:898092 | CTGF | Connective tissue growth factor | NM_001901 |
| 6 | IMAGE:526184 | RAB21 |  |  |
| 7 | 1292741 | EST | Transcribed locus | AA719374 |
| 8 | IMAGE:526657 | TCEB3 | Transcription elongation factor B (SIII), polypeptide 3 (110kDa, elongin A) | AK096079 |
| 9 | IMAGE:701602 | EPB41L5 |  |  |
| 10 | IMAGE:759163 | MFAP4 | Microfibrillar-associated protein 4 | BC035560 |
| 11 | IMAGE:277305 | RHOB | Ras homolog gene family, member B | NM_004040 |
| 12 | IMAGE:51448 | ATF3 | Activating transcription factor 3 | NM_001040619 |
| 13 | IMAGE:433491 | MYADM | Myeloid-associated differentiation marker | NM_001020818 |
| 14 | IMAGE:767469 | ANKH |  |  |
| 15 | IMAGE:300973 | CD8A | CD8a molecule | AK124156 |
| 16 | IMAGE:1736819 | UACA | Uveal autoantigen with coiled-coil domains and ankyrin repeats | NM_001008224 |
| 17 | IMAGE:486055 | CYP1B1 | Cytochrome P450, family 1, subfamily B, polypeptide 1 | NM_000104 |
| 18 | IMAGE:1508768 | TMEM16B | Transmembrane protein 16B | NM_020373 |
| 19 | IMAGE:346860 | EST | Transcribed locus | BF673688 |
| 20 | IMAGE:50562 | C8orf4 | Chromosome 8 open reading frame 4 | NM_020130 |
| 21 | IMAGE:795288 | USP4 | Ubiquitin specific peptidase 4 (proto-oncogene) | NM_003363 |
| 22 | IMAGE:359610 | BMP2 | Bone morphogenetic protein 2 | NM_001200 |
| 23 | IMAGE:811953 | MCM4 |  |  |
| 24 | IMAGE:489495 | MYADM | Myeloid-associated differentiation marker | NM_001020818 |
| 25 | IMAGE:503155 | AP4E1 | Adaptor-related protein complex 4, epsilon 1 subunit | NM_007347 |
| 26 | IMAGE:268652 | CDKN1A | Cyclin-dependent kinase inhibitor 1A (p21, Cip1) | NM_078467 |
| 27 | IMAGE:866633 | BTBD3 | BTB (POZ) domain containing 3 | NM_014962 |
| 28 | IMAGE:132911 | PPP1CB | Protein phosphatase 1, catalytic subunit, beta isoform | BQ278323 |
| 29 | IMAGE:261444 | MYADM | Myeloid-associated differentiation marker | NM_001020818 |
| 30 | IMAGE:397488 | TBX3 | T-box 3 (ulnar mammary syndrome) | NM_016569 |
| 31 | IMAGE:150216 | KIAA1337 |  |  |
| 32 | IMAGE:296141 | KBTBD2 | Kelch repeat and BTB (POZ) domain containing 2 | AB040922 |
| 33 | IMAGE:786657 | CYP1B1 | Cytochrome P450, family 1, subfamily B, polypeptide 1 | NM_000104 |
| 34 | IMAGE:366889 | KRT17 | Keratin 17 | BX647923 |
| 35 | IMAGE:868472 | CLIC4 | Transcribed locus | BM994958 |
| 36 | IMAGE:194986 | RBBP6 | Retinoblastoma binding protein 6 | NM_006910 |
| 37 | IMAGE:868630 | TSC22D1 | TSC22 domain family, member 1 | NM_183422 |
| 38 | IMAGE:753381 | SEC23A | Sec23 homolog A (S. cerevisiae) | BC036649 |
| 39 | IMAGE:814792 | USP10 | Ubiquitin specific peptidase 10 | NM_005153 |
| 40 | IMAGE:46011 | TBC1D24 | TBC1 domain family, member 24 | BX648283 |
| 41 | IMAGE:48182 | PGM5 | Transcribed locus, strongly similar to NP_068800.1 5 [Homo sapiens] | CD559141 |
| 42 | IMAGE:79848 | NEDD9 | Neural precursor cell expressed, developmentally down-regulated 9 | BX648041 |
| 43 | IMAGE:824694 | PTP4A1 | Protein tyrosine phosphatase type IVA, member 1 | NM_003463 |
| 44 | IMAGE:142184 | GOLT1B | Golgi transport 1 homolog B (S. cerevisiae) | AB097020 |
| 45 | IMAGE:35311 | AKAP5 | A kinase (PRKA) anchor protein 5 | AK124004 |
| 46 | IMAGE:41029 | CLASP1 | Cytoplasmic linker associated protein 1 | NM_015282 |
| 47 | IMAGE:591265 | KRT17 | Keratin 17 | BX647923 |
| 48 | IMAGE:290263 | EST | Transcribed locus | BQ010713 |
| 49 | IMAGE:230613 | EST | Transcribed locus | H75478 |
| 50 | IMAGE:810724 | IER3 | Immediate early response 3 |  |
| 51 | IMAGE:300289 | CIB2 | Calcium and integrin binding family member 2 | NM_006383 |
| 52 | IMAGE:795820 | SPINK5L3 | Serine PI Kazal type 5-like 3 | AK001520 |
| 53 | IMAGE:897562 | GABPA | GA binding protein transcription factor, alpha subunit 60kDa | BX647755 |
| 54 | IMAGE:2013515 | SGK | Serum/glucocorticoid regulated kinase | BX649005 |
| 55 | IMAGE:396045 | AASDHPPT | Aminoadipate-semialdehyde dehydrogenase-phosphopantetheinyl transferase | BX537665 |
| 56 | IMAGE:878182 | A2M | Alpha-2-macroglobulin | CR749334 |
| 57 | IMAGE:826350 | GPS1 | G protein pathway suppressor 1 | AB209596 |
| 58 | IMAGE:949944 | CASP2 | Transcribed locus | BG387557 |
| 59 | IMAGE:701371 | TNPO1 | Transportin 1 | NM_002270 |
| 60 | IMAGE:1456721 | MRPL42 | Mitochondrial ribosomal protein L42 | CR749344 |
| 61 | IMAGE:840708 | SOD2 | Superoxide dismutase 2, mitochondrial | AK097395 |
| 62 | IMAGE:294995 | PAFAH1B2 | Platelet-activating factor acetylhydrolase, isoform Ib, beta subunit 30kDa | BC021287 |
| 63 | IMAGE:28469 | OXCT1 | 3-oxoacid CoA transferase 1 | NM_000436 |
| 64 | IMAGE:142022 | ANKRD57 | Ankyrin repeat domain 57 | NM_023016 |
| 65 | IMAGE:705110 | CASP9 | Caspase 9, apoptosis-related cysteine peptidase | AB209147 |
| 66 | IMAGE:811048 | PLXND1 | Plexin D1 | NM_015103 |
| 67 | IMAGE:814329 | DXF68S1E |  |  |
| 68 | IMAGE:42739 | PTP4A1 | Protein tyrosine phosphatase type IVA, member 1 | NM_003463 |
| 69 | IMAGE:284306 | FBXL3 | F-box and leucine-rich repeat protein 3 | AL833187 |
| 70 | IMAGE:22908 | EST | Transcribed locus | CD518535 |
| 71 | IMAGE:301995 | SORBS2 | Sorbin and SH3 domain containing 2 | NM_021069 |
| 72 | IMAGE:256983 | DDX55 | DEAD (Asp-Glu-Ala-Asp) box polypeptide 55 | AB046815 |
| 73 | IMAGE:1741810 | EST | Transcribed locus | CN296089 |
| 74 | IMAGE:26505 | DPYSL5 | Dihydropyrimidinase-like 5 | AL713706 |
| 75 | IMAGE:726597 | VPS37A | Vacuolar protein sorting 37 homolog A (S. cerevisiae) | AL834189 |
| 76 | IMAGE:364685 | CENTB5 | Centaurin, beta 5 | AB051503 |
| 77 | IMAGE:360254 | CYR61 | Cysteine-rich, angiogenic inducer, 61 | Y11307 |
| 78 | IMAGE:23461 | SLCO3A1 | Solute carrier organic anion transporter family, member 3A1 | AF205074 |
| 79 | IMAGE:257960 | CHORDC1 | Cysteine and histidine-rich domain (CHORD)-containing 1 | BX537692 |
| 80 | IMAGE:811918 | BTBD3 |  |  |
| 81 | IMAGE:753467 | SLC2A3 | Solute carrier family 2 (facilitated glucose transporter), member 3 | AB209607 |
| 82 | IMAGE:1684695 | CCDC132 | Coiled-coil domain containing 132 | NM_017667 |
| 83 | IMAGE:753907 | PHLDB2 | Pleckstrin homology-like domain, family B, member 2 | AL832205 |
| 84 | IMAGE:188232 | KLF4 | Kruppel-like factor 4 (gut) | NM_004235 |
| 85 | IMAGE:868652 | C4B | Complement component 4B (Childo blood group) | NM_001002029 |
| 86 | IMAGE:1566915 | GRIP1 | Glutamate receptor interacting protein 1 | XM_001133925 |
| 87 | IMAGE:246449 | IRF2BP2 | Interferon regulatory factor 2 binding protein 2 | NM_182972 |
| 88 | IMAGE:143790 | OGT | O-linked N-acetylglucosamine (GlcNAc) transferase | AL050366 |
| 89 | IMAGE:52076 | OLFM1 | Olfactomedin 1 | NM_014279 |
| 90 | IMAGE:511778 | TM4SF1 | Transmembrane 4 L six family member 1 | AL832780 |
| 91 | IMAGE:898076 | IRF2BP2 | Interferon regulatory factor 2 binding protein 2 | NM_182972 |
| 92 | IMAGE:814319 | CHPPR |  |  |
| 93 | IMAGE:768059 | C6orf62 | Chromosome 6 open reading frame 62 | AL136632 |
| 94 | IMAGE:825369 | VGLL4 | Vestigial like 4 (Drosophila) | AK126479 |
| 95 | IMAGE:1503811 | EST | Transcribed locus | BX093604 |
| 96 | IMAGE:272238 | FAM91A1 | Family with sequence similarity 91, member A1 | AL832999 |
| 97 | IMAGE:815503 | RCOR1 | REST corepressor 1 | NM_015156 |
| 98 | IMAGE:504187 | TMF1 | TATA element modulatory factor 1 | NM_007114 |
| 99 | IMAGE:813533 | SDCBP | Syndecan binding protein (syntenin) | AK128645 |
| 100 | IMAGE:1738269 | EST | Transcribed locus | AL119005 |
| 101 | IMAGE:753381 | SEC23A | Sec23 homolog A (S. cerevisiae) | BC036649 |

Table S2: List of 100 genes with highest expression at any time point (*Shake 2*)
