# Supplementary material for: Identification of G1-Regulated Genes in Normally Cycling Human Cells
Source: PLoS One. 2008 Dec 15;3(12):e3943. doi: 10.1371/journal.pone.0003943 (PMC2600614; doi:10.1371/journal.pone.0003943)
Supplement: Table S3 — List of 100 genes with lowest expression at any time point (Shake 2). Genome-scale analysis of G1-regulated genes. The identified genes are presented using their corresponding clone IDs. Gene names and accession numbers displayed in all tables were generated from the SMD online analysis software (http://genome-www5.stanford.edu/), and accession numbers were further verified using the S.O.U.R.C.E online tool (http://genome-www5.stanford.edu/cgi-bin/source/sourceSearch). The full data is available online (http://www.ncbi.nlm.nih.gov/geo/query/acc.cgi?accGSE12473) (0.14 MB DOC) [file pone.0003943.s003.doc]

| **#** | **CloneID** | **Genesymbol** | **Genename** | **Acc. Num.** |
| --- | --- | --- | --- | --- |
| 1 | IMAGE:2017930 | CIB2 | Calcium and integrin binding family member 2 | NM_006383 |
| 2 | IMAGE:769921 | UBE2C | Ubiquitin-conjugating enzyme E2C | BC032677 |
| 3 | IMAGE:30170 | CASP3 | Caspase 3, apoptosis-related cysteine peptidase | NM_004346 |
| 4 | IMAGE:241043 | TncRNA | Trophoblast-derived noncoding RNA | EF177379 |
| 5 | IMAGE:1734282 | EST | Transcribed locus | AI183564 |
| 6 | IMAGE:868077 | EST | Transcribed locus | AA780530 |
| 7 | IMAGE:1658249 | COBL | Cordon-bleu homolog (mouse) | AB014533 |
| 8 | IMAGE:1732811 | TULP3 | Tubby like protein 3 | NM_003324 |
| 9 | IMAGE:859450 | LIN7B | Lin-7 homolog B (C. elegans) | BG749971 |
| 10 | IMAGE:49318 | AXL | AXL receptor tyrosine kinase | NM_021913 |
| 11 | IMAGE:970391 | PCP4L1 | Purkinje cell protein 4 like 1 | BC028905 |
| 12 | IMAGE:995512 | PGAM5 | Phosphoglycerate mutase family member 5 | AK097688 |
| 13 | IMAGE:25063 | MAP7D2 | MAP7 domain containing 2 | BC089400 |
| 14 | IMAGE:108837 | CCL2 | Chemokine (C-C motif) ligand 2 | BU570769 |
| 15 | IMAGE:771277 | YWHAZ | Tyrosine 3-monooxygenase/tryptophan 5-monooxygenase activation protein, zeta polypeptide | BC051814 |
| 16 | IMAGE:1587791 | LRRC61 | Leucine rich repeat containing 61 | AK127403 |
| 17 | IMAGE:1622265 | EST | Transcribed locus, moderately similar to XP_117451.1 hypothetical protein XP_117451 [Homo sapiens] | CF995058 |
| 18 | IMAGE:146882 | UBE2C | Ubiquitin-conjugating enzyme E2C | BC032677 |
| 19 | IMAGE:1741607 | NAB1 | NGFI-A binding protein 1 (EGR1 binding protein 1) | XM_001129667 |
| 20 | IMAGE:1700916 | CNTLN | Centlein, centrosomal protein | BX647069 |
| 21 | IMAGE:160838 | SMARCC2 | SWI/SNF related, matrix associated, actin dependent regulator of chromatin, subfamily c, member 2 | AB209006 |
| 22 | IMAGE:969311 | EST | Transcribed locus | AA663638 |
| 23 | IMAGE:152470 | LRCH4 | Leucine-rich repeats and calponin homology (CH) domain containing 4 | NM_002319 |
| 24 | IMAGE:859786 | RAPGEF1 | Rap guanine nucleotide exchange factor (GEF) 1 | NM_198679 |
| 25 | IMAGE:1007830 | EST | Transcribed locus | AI820955 |
| 26 | IMAGE:129858 | ANLN | Anillin, actin binding protein | NM_018685 |
| 27 | IMAGE:1627154 | C11orf17 | Chromosome 11 open reading frame 17 | AK075308 |
| 28 | IMAGE:1858830 | EST | Transcribed locus | AI202772 |
| 29 | IMAGE:1008639 | EST | Transcribed locus | AL119103 |
| 30 | IMAGE:811936 | RNF31 | Ring finger protein 31 | NM_017999 |
| 31 | IMAGE:359285 | CPA4 | Carboxypeptidase A4 | NM_016352 |
| 32 | IMAGE:292219 | CFI | Complement factor I | AK122686 |
| 33 | IMAGE:1624720 | EST | Transcribed locus | AA993698 |
| 34 | IMAGE:897007 | ZC3H7B | Zinc finger CCCH-type containing 7B | NM_017590 |
| 35 | IMAGE:1593585 | EST | Transcribed locus | AI733425 |
| 36 | IMAGE:1603676 | MTX3 | Metaxin 3 | BX647596 |
| 37 | IMAGE:180657 |  | MRNA; cDNA DKFZp547A0515 (from clone DKFZp547A0515) | AL831835 |
| 38 | IMAGE:867606 | COL8A2 | Collagen, type VIII, alpha 2 | NM_005202 |
| 39 | IMAGE:1574513 | EST | Transcribed locus | CA414792 |
| 40 | IMAGE:1858306 | BAHCC1 | BAH domain and coiled-coil containing 1 | NM_001080519 |
| 41 | IMAGE:233712 | PPP1R3E | Protein phosphatase 1, regulatory (inhibitor) subunit 3E | XM_927029 |
| 42 | IMAGE:771891 | EST | Transcribed locus | AI732711 |
| 43 | IMAGE:898124 |  |  |  |
| 44 | IMAGE:1604340 | EST | Transcribed locus | AV758315 |
| 45 | IMAGE:969636 | SNN | Stannin | NM_003498 |
| 46 | IMAGE:1155467 | EST | Transcribed locus | AA678525 |
| 47 | IMAGE:272711 | CDC42BPA | CDC42 binding protein kinase alpha (DMPK-like) | NM_003607 |
| 48 | IMAGE:1623764 | MGC39606 | Hypothetical protein MGC39606 | BC030620 |
| 49 | IMAGE:951230 | SNAPC3 | Small nuclear RNA activating complex, polypeptide 3, 50kDa | NM_001039697 |
| 50 | IMAGE:213585 | EST | Transcribed locus | BX096294 |
| 51 | IMAGE:1585327 | AXIN2 | Axin 2 (conductin, axil) | CR933657 |
| 52 | IMAGE:1871085 | SCAMP4 | Secretory carrier membrane protein 4 | AK091166 |
| 53 | IMAGE:854425 | PGPEP1 | Pyroglutamyl-peptidase I | NM_017712 |
| 54 | IMAGE:1007795 | EST | Transcribed locus | CN264199 |
| 55 | IMAGE:283933 | SLTM | SAFB-like, transcription modulator | NM_024755 |
| 56 | IMAGE:285992 | ADNP | Activity-dependent neuroprotector | BC075794 |
| 57 | IMAGE:1591154 | SAR1A | SAR1 gene homolog A (S. cerevisiae) | NM_020150 |
| 58 | IMAGE:2018586 | DLL1 | Delta-like 1 (Drosophila) | NM_005618 |
| 59 | IMAGE:1555537 | PLCXD1 | Phosphatidylinositol-specific phospholipase C, X domain containing 1 | AK091006 |
| 60 | IMAGE:852947 | EST | Transcribed locus | AA668205 |
| 61 | IMAGE:1926364 | RBM9 | RNA binding motif protein 9 | NM_001031695 |
| 62 | IMAGE:666084 | TIMP4 |  |  |
| 63 | IMAGE:530875 | TKT | Transketolase (Wernicke-Korsakoff syndrome) | BX649193 |
| 64 | IMAGE:392093 | LASP1 | LIM and SH3 protein 1 | NM_006148 |
| 65 | IMAGE:950271 | HCMOGT-1 |  |  |
| 66 | IMAGE:1471451 | PLEKHB1 | Pleckstrin homology domain containing, family B (evectins) member 1 | U89715 |
| 67 | IMAGE:180855 | PNCK | Pregnancy upregulated non-ubiquitously expressed CaM kinase | BC064422 |
| 68 | IMAGE:1686735 |  | CDNA clone IMAGE:5300185 | BC039399 |
| 69 | IMAGE:842846 | TIMP2 | TIMP metallopeptidase inhibitor 2 | NM_003255 |
| 70 | IMAGE:1859319 | FLJ39632 | Hypothetical LOC642477 | AK096951 |
| 71 | IMAGE:1551395 | EST | Transcribed locus | AA934016 |
| 72 | IMAGE:345616 | GPNMB | Glycoprotein (transmembrane) nmb | BC032783 |
| 73 | IMAGE:1926575 | CDX2 | Caudal type homeobox transcription factor 2 | NM_001265 |
| 74 | IMAGE:1471675 | BTBD5 |  |  |
| 75 | IMAGE:1684274 | THRB | Thyroid hormone receptor, beta (erythroblastic leukemia viral (v-erb-a) oncogene homolog 2, avian) | NM_000461 |
| 76 | IMAGE:1670927 | ZNF688 | Zinc finger protein 688 | AK122680 |
| 77 | IMAGE:2029256 | EST | Transcribed locus | BU627449 |
| 78 | IMAGE:278570 | MITF |  |  |
| 79 | IMAGE:1699067 | EST | Transcribed locus | AI792334 |
| 80 | IMAGE:826178 | CCDC18 | Coiled-coil domain containing 18 | NM_206886 |
| 81 | IMAGE:1559002 | C6orf166 | Chromosome 6 open reading frame 166 | CR599380 |
| 82 | IMAGE:1571106 | JMJD2B | Jumonji domain containing 2B | AB020683 |
| 83 | IMAGE:147133 | EST | Transcribed locus | CB242575 |
| 84 | IMAGE:1700327 | EST | Transcribed locus | AI049660 |
| 85 | IMAGE:1473118 | SLC6A19 | Solute carrier family 6 (neutral amino acid transporter), member 19 | AK096054 |
| 86 | IMAGE:954072 | EST | Transcribed locus | BF110518 |
| 87 | IMAGE:80357 | P2RY1 | Purinergic receptor P2Y, G-protein coupled, 1 | NM_002563 |
| 88 | IMAGE:247559 | C14orf43 | Chromosome 14 open reading frame 43 | NM_194278 |
| 89 | IMAGE:278243 | ARHGAP11B | Rho GTPase activating protein 11B | BC071990 |
| 90 | IMAGE:781088 | RP11-151A6.2 | Hypothetical protein BC004360 | AK095850 |
| 91 | IMAGE:1586142 | OSBPL10 | Oxysterol binding protein-like 10 | AB209872 |
| 92 | IMAGE:80162 | RAD51C | RAD51 homolog C (S. cerevisiae) | BC073161 |
| 93 | IMAGE:26289 | EHMT1 | Euchromatic histone-lysine N-methyltransferase 1 | AB058779 |
| 94 | IMAGE:884660 | DXS542 | Putative X-linked retinopathy protein | AK129657 |
| 95 | IMAGE:179220 | PHYH | Phytanoyl-CoA 2-hydroxylase | CR623416 |
| 96 | IMAGE:1008356 | EST | Transcribed locus | AL119103 |
| 97 | IMAGE:566887 | CBX3 | Chromobox homolog 3 (HP1 gamma homolog, Drosophila) | BX647444 |
| 98 | IMAGE:454538 | TTC9C | Tetratricopeptide repeat domain 9C | AF289605 |
| 99 | IMAGE:190468 | NR4A3 | Nuclear receptor subfamily 4, group A, member 3 | NM_173198 |
| 100 | IMAGE:1609538 | KIAA1324 | KIAA1324 | AB037745 |
| 101 | IMAGE:1558505 | LEPRE1 | Leucine proline-enriched proteoglycan (leprecan) 1 | NM_022356 |

Table S3: List of 100 genes with lowest expression at any time point (*Shake 2*)
