# Supplementary material for: Identification of G1-Regulated Genes in Normally Cycling Human Cells
Source: PLoS One. 2008 Dec 15;3(12):e3943. doi: 10.1371/journal.pone.0003943 (PMC2600614; doi:10.1371/journal.pone.0003943)
Supplement: Table S4 — GO analysis. Gene ontology analysis of G1 genes (see supplemental Table S1). Columns represent GO “biological process” category, number of genes under that category (# of Genes), representation in percent (%) to total number of genes (127, output of DAVID analysis), p-value, fold enrichment and false discovery rate (FDR<10). For further information see methods section. (0.05 MB DOC) [file pone.0003943.s004.doc]

| **GO Term** | **# of Genes** | **%** | **PValue** | **Enrichment** | **FDR** |
| --- | --- | --- | --- | --- | --- |
| Ras protein signal transduction | 9 | 7 | 1.38E-04 | 5.9 | 0.3 |
| Regulation of Ras protein signal transduction | 8 | 6 | 1.45E-04 | 6.9 | 0.3 |
| Intracellular signaling cascade | 21 | 17 | 2.90E-04 | 2.4 | 0.6 |
| Regulation of small GTPase mediated signal transduction | 8 | 6 | 5.47E-04 | 5.6 | 1.0 |
| Cell morphogenesis | 11 | 9 | 6.11E-04 | 3.8 | 1.2 |
| Cellular structure morphogenesis | 11 | 9 | 6.11E-04 | 3.8 | 1.2 |
| Cell growth | 7 | 6 | 8.68E-04 | 6.2 | 1.6 |
| Regulation of cell size | 7 | 6 | 9.91E-04 | 6.1 | 1.9 |
| Calcium-mediated signaling | 4 | 3 | 0.001267111 | 18.5 | 2.4 |
| Regulation of cell growth | 6 | 5 | 0.002003191 | 6.6 | 3.8 |
| Negative regulation of cellular process | 16 | 13 | 0.002039172 | 2.4 | 3.8 |
| Regulation of catalytic activity | 10 | 8 | 0.002048071 | 3.5 | 3.8 |
| Protein processing | 5 | 4 | 0.002704589 | 8.5 | 5.0 |
| Negative regulation of biological process | 16 | 13 | 0.003067548 | 2.3 | 5.7 |
| Protein amino acid autophosphorylation | 4 | 3 | 0.00357382 | 12.9 | 6.6 |
| Protein autoprocessing | 4 | 3 | 0.003969206 | 12.4 | 7.3 |
| Blood vessel development | 6 | 5 | 0.004716504 | 5.4 | 8.6 |
| Regulation of a molecular function | 10 | 8 | 0.004746453 | 3.1 | 8.7 |
| Vasculature development | 6 | 5 | 0.005040946 | 5.4 | 9.2 |
| Cell cycle | 13 | 10 | 0.005323897 | 2.5 | 9.7 |
| Anatomical structure morphogenesis | 15 | 12 | 0.005426813 | 2.3 | 9.9 |

Table S4: GO analysis of G1-regulated genes (see supplemental Table S1)
